# Adipose tissue arachidonic acid content is associated with the expression of 5-lipoxygenase in atherosclerotic plaques

**DOI:** 10.1186/1476-511X-12-7

**Published:** 2013-01-25

**Authors:** Michael S Nielsen, Marie-Louise M Grønholdt, Mogens Vyberg, Kim Overvad, Annette Andreasen, Karen-Margrete Due, Erik B Schmidt

**Affiliations:** 1 Department of Cardiology, Center for Cardiovascular Research, Aalborg University Hospital, Soendre Skovvej 15, 9000, Aalborg, Denmark; 2 Department of Surgery, Nyborg Hospital, Vestergade 17, 5800, Nyborg, Denmark; 3 Institute of Pathology, Aalborg University Hospital, Ladegaardsgade 3, 9100, Aalborg, Denmark; 4 Section for Epidemiology, Department of Public Health, Aarhus University, Bartholin Allé 2, 8000, Aarhus, Denmark

**Keywords:** Adipose tissue, Arachidonic acid, Eicosapentaenoic acid, Atherosclerotic plaques, 5-Lipoxygenase, Leukotriene B_4_, Cysteinyl leukotrienes

## Abstract

**Background:**

The content of arachidonic acid in adipose tissue is positively associated with the risk of myocardial infarction, whereas the content of eicosapentaenoic acid in adipose tissue has been reported to be negatively associated with the risk of myocardial infarction. Both arachidonic acid and eicosapentaenoic acid are substrates for the synthesis of pro-inflammatory leukotrienes and leukotrienes derived from eicosapentaenoic acid are generally much less potent. In this study we hypothesized that a high content of arachidonic acid in adipose tissue would reflect a high formation of arachidonic acid derived leukotrienes and a high expression of 5-lipoxygenase in atherosclerotic plaques. Likewise, we hypothesized that a high content of eicosapentaenoic acid in adipose tissue would reflect a low formation of arachidonic acid derived leukotrienes and a low expression of 5-lipoxygenase in plaques.

**Methods:**

In a cross sectional study we included 45 consecutive subjects undergoing femoral thrombendarterectomy. The expression of 5-lipoxygenase in plaques was assessed by a semi-automated image analysis computer programme after immunohistochemical staining with mono-clonal 5-lipoxygenase antibodies. Leukotriene B_4_ and cysteinyl leukotriene formation from stimulated femoral artery plaques was quantified using ELISA methods. The fatty acid content of adipose tissue biopsies from the thigh was analyzed using gas chromatography. Associations between variables were assessed by Pearson correlations and were further explored in a multivariable linear regression model adjusting for potential confounders.

**Results:**

A high content of arachidonic acid in adipose tissue was associated with a higher expression of 5-lipoxygenase in plaques (r = 0.32, p = 0.03), but no significant associations with leukotriene B_4_ (r = 0.22, p = 0.14) and cysteinyl leukotriene (r = −0.11, p = 0.46) formation was seen. No significant associations were found between the content of eicosapentaenoic acid in adipose tissue and 5-lipoxygenase expression or leukotriene formation in plaque.

**Conclusions:**

Adipose tissue arachidonic acid contents correlated positively with the expression of 5-lipoxygenase in plaques. This association might represent a causal link between adipose tissue arachidonic acid and the risk of myocardial infarction but confirmatory studies are needed.

## Background

**Figure 1:**
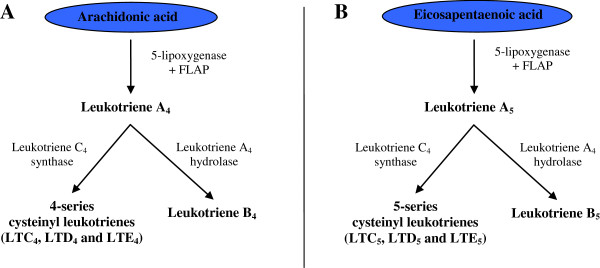
**Formation of leukotrienes from arachidonic acid and eicosapentaenoic acid.** To the left is seen the synthesis of 4-series leukotrienes derived from arachidonic acid (ARA). The initial step is catalyzed by 5-lipoxygenase and leukotriene A_4_ is formed. Next, depending on enzyme availability of the individual cell, either leukotriene B_4_ or leukotriene C_4_ will be formed. If leukotriene C_4_ is formed, it is quickly converted to leukotriene D_4_ and then to leukotriene E_4_, and these leukotrienes are collectively known as cysteinyl leukotrienes. To the right is seen the synthesis of 5-series leukotrienes derived from eicosapentaenoic acid (EPA). Since EPA is chemically identical to ARA, apart from one additional double bond, it serves as a co-substrate for the enzymes used for the formation of 4-series leukotrienes (competitive inhibitor).

The marine omega-3 polyunsaturated fatty acid (PUFA), eicosapentaenoic acid (EPA) is generally considered to be beneficial in the prevention of coronary artery disease [1] whereas the n-6 PUFA, arachidonic acid (ARA), has been linked to a higher risk [2-4]. Both ARA and EPA serve as substrates for 5-lipoxygenase, the initiating enzyme in the biosynthesis of pro-inflammatory leukotrienes (Figure 1) and as described below, the leukotrienes derived from EPA are generally much less potent as compared to those derived from ARA. The ARA-derived leukotrienes have been linked to the development of atherosclerosis and plaque instability via increased leukocyte chemotaxis, vascular inflammation and subsequent matrix degeneration [5]. Leukotriene B_4_ (LTB_4_) is one of the most potent chemotactic agents known to date, and the cysteinyl leukotrienes (LTC_4_, LTD_4_ and LTE_4_) are powerful spasmogenic agents in asthma. ARA is the natural precursor of the above mentioned 4-series (i.e. 4 double bonds) leukotrienes formed in activated leukocytes, including macrophages. On the other hand, EPA can act as an alternative substrate, leading to the formation of 5-series leukotrienes (LTB_5_, LTC_5_, LTD_5_, LTE_5_) of which LTB_5_ is at least 30 times less potent in chemoattractant and aggregating properties compared to the ARA-derived LTB_4_[6]. The cysteinyl leukotrienes, however, seem to possess equally potent biological activities regardless of the number of double bonds [7,8].

Adipose tissue is widely used in epidemiological studies as a biomarker of long term intake of PUFA [9]. A high adipose tissue content of marine omega-3 PUFA has been associated with a lower risk of acute coronary syndrome [10,11], while on the contrary, a high content of ARA in adipose tissue has been associated with a higher risk of myocardial infarction [2-4]. The mechanistic explanation of these findings is unsettled, but impacts on plaque inflammation and thus plaque stability could be an important factor. In the present study, we hypothesized that the content of EPA in adipose tissue reflect lower levels of 5-lipoxygenase and 4-series leukotrienes in femoral atherosclerotic plaques, and that the content of ARA in adipose tissue, on the other hand, is associated with higher levels of 4-series leukotrienes and 5-lipoxygenase in plaques. Since EPA and ARA in adipose tissue must be liberated into the circulation and probably also incorporated into atherosclerotic plaques in order to affect the lipoxygenase pathway in the arterial wall, our secondary aim was to assess the correlation between the content of these fatty acids in adipose tissue, plasma, and atherosclerotic femoral plaques, respectively.

## Results

### Subject characteristics

Fifty subjects undergoing femoral thrombendarterectomy were included in the study but five subjects were later withdrawn from the study due to delays in surgery, caused by non-study related medical emergencies in other patients. These delays entailed logistic problems, for which reason atherosclerotic plaques and/or blood samples were not collected for this study from these five patients. Subject characteristics are given in Table 1. The median age of the 45 subjects included in the final analysis was 71 years, and both sexes were equally represented (53% males). More than half (58%) had symptomatic atherosclerotic disease from more than one arterial vascular bed. The median intake of marine omega-3 polyunsaturated fatty acids (EPA + DHA) was 0.68 g/day.

**Table 1:** Baseline characteristics

**Variable**	**Value**
Age*, years	71 (54 – 81)
Sex (% men)	53
Body mass index*, kg/m^2^	25 (18 – 30)
EPA + DHA intake* (g/day)	0.68 (0.16 – 1.69)
Current smokers, %	33
**Medical history and medications**	
Cardiovascular disease†, %	58
Diabetes mellitus, %	24
Aspirin treatment, %	98
Anti-hypertensive medication, %	78
Statin treatment, %	96
NSAID use^‡,^ %	7
**Laboratory examination**	
Total cholesterol*, mmol/l	3.8 (3.1 – 4.6)
P-glucose*, mmol/l	5.9 (5.3 – 8.7)
P-hsCRP^§^, mg/l (n = 37)^e^	1.8 (0.3 – 6.4)
P-creatinine*, μmol/l	85 (56 – 107)

* Median (10^th^ and 90^th^ percentiles).

† Any diagnosis of stable angina, unstable angina, MI, ischemic stroke, transitory ischemic attack or any procedure of percutaneus coronary intervention or coronary artery bypass grafting.

^‡^ Chronic use of non-steroidal anti-inflammatory drugs (NSAID).

^§^ Subjects with hsCRP > 10 mmol/l (n = 8) were omitted since these values suggest acute rather than low-grade inflammation.

### Plaque characteristics

Atherosclerotic plaques were all advanced lesions. They were generally highly fibrotic and heavily calcified and 71% were without any lipid core. Inflammatory cells were present in very low numbers. The median expression of 5-lipoxygenase in the plaques was 0.19% and median LTB_4_ and cysteinyl leukotriene formation from stimulated plaques were 3.4 ng/g and 7.3 ng/g, respectively. A statistically significant correlation between 5-lipoxygenase expression and LTB_4_ formation (r = 0.32, 95% CI: 0.03; 0.56, p = 0.03), but not cysteinyl leukotriene formation (r = 0.06, 95% CI: -0.24; 0.35, p = 0.71), was observed.

### Associations between the content of ARA and EPA in adipose tissue and the expression of 5-lipoxygenase and formation of leukotrienes in plaques

Adipose tissue ARA was positively correlated with both the expression of 5-lipoxygenase in plaques (r = 0.32) and the formation of LTB_4_ in plaques, (r = 0.22), although the latter was not statistically significant (Table 2). No significant association between adipose tissue ARA content and formation cysteinyl leukotrienes in plaques were observed. The content of EPA in adipose tissue was positively, but not significantly, correlated with expression of 5-lipoxygenase (r = 0.14), formation of LTB_4_ (r = 0.25), and formation of cysteinyl leukotrienes (r = 0.02). Neither the content of ARA nor the content of EPA in adipose tissue was significantly correlated with LTE_4_ levels in urine (a marker of total body cysteinyl leukotriene formation). The association between the content of ARA in adipose tissue and 5-lipoxygenase was further explored in a linear regression model, mainly to elucidate whether adjustment for age, sex, and BMI would change the conclusions. In the crude model, the regression coefficient for the association between the content of ARA in adipose tissue and 5-lipoxygenase expression in plaque was 0.35% (95% CI: 0.06; 0.64, p = 0.019) and the regression coefficient was 0.47% (95% CI: 0.16; 0.78, p = 0.004) after adjustment for age, sex and BMI. No statistically significant associations were found in any of the other linear regression analyses, and adjustment for age, sex, and BMI (data not shown) did not change any of the conclusions already obtained from the correlation analyses.

**Table 2:** Correlations between PUFA content in adipose tissue and 5-lipoxygenase pathway constituents in plaque and urine

	**Adipose tissue arachidonic acid (% of total fatty acids)**			**Adipose tissue eicosapentaenoic acid (% of total fatty acids)**		
	**r**	**95% CI**	**p**	**r**	**95% CI**	**p**
**Plaque**						
5-lipoxygenase expression (% of plaque area)	0.32	0.03 to 0.56	0.03	0.14	−0.16 to 0.41	0.37
Leukotriene B_4_ (ng/g)	0.22	−0.08 to 0.42	0.14	0.25	−0.05 to 0.50	0.10
Cysteinyl leukotrienes (ng/g)	−0.11	−0.39 to 0.19	0.46	0.02	−0.27 to 0.31	0.87
**Urine**						
Leukotriene E_4_ (pg/mg creatinine)	0.02	−0.28 to 0.31	0.92	−0.12	−0.40 to 0.18	0.44

### Associations between the content of linoleic acid and docosahexaenoic acid in adipose tissue and the expression of 5-lipoxygenase and formation of leukotrienes in plaques

Since both linoleic acid and docosahexaenoic acid could indirectly affect the content of ARA and EPA in adipose tissue and plaques, we included these PUFA in an exploratory analysis. No substantial nor statistically significant correlations were observed between the content of docosahexaenoic acid in adipose tissue and 5-lipoxygenase expression in plaque (r = 0.04, 95% CI: -0.26; 0.33, p = 0.80), LTB_4_ in plaques (r = 0.18, 95% CI: -0.12; 0.45, p = 0.23), cysteinyl leukotrienes in plaques (r = 0.01, 95% CI: -0.28; 0.30, p = 0.96) or urine LTE_4_ levels (r = −0.04, 95% CI: -0.34; 0.25, p = 0.75). Likewise, the content of linoleic acid in adipose tissue was neither substantially nor statistically significantly correlated with 5-lipoxygenase expression in plaque (r = −0.06, 95% CI: -0.35; 0.24, p = 0.70), LTB_4_ in plaques (r = −0.09, 95% CI: -0.37; 0.21, p = 0.55), cysteinyl leukotrienes in plaques (r = 0.09, 95% CI: -0.21; 0.37, p = 0.57), or urine LTE_4_ levels (r = −0.05, 95% CI: -0.34; 0.25, p = 0.73).

### Correlations between the content of EPA and ARA in adipose tissue, plasma, and atherosclerotic plaques

**Figure 2:**
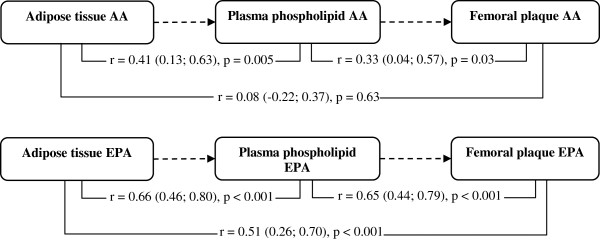
**Schematic of the correlations between adipose tissue, plasma phospholipid and plaque content of arachidonic acid and eicosapentaenoic acid. A:** Positive correlations were found between the content of arachidonic acid (ARA) in adipose tissue and plasma phospholipids as well as between plasma phospholipids and atherosclerotic plaques. No significant correlation between the content of ARA in adipose tissue and plaque was observed. **B:** Strong positive correlations between the content of eicosapentaenoic acid (EPA) in all three compartments were found indicating that EPA is readily incorporated into all these compartments.

The median content of ARA and EPA was 0.34% and 0.09% in adipose tissue, 10.74% and 1.91% in plasma phospholipids, and 6.45% and 0.81% in plaque tissue. As can be seen from Figure 2, we observed statistically significant positive correlations, both between ARA in adipose tissue and ARA in plasma phospholipids, and between ARA in plasma phospholipids and ARA in plaques. ARA in adipose tissue and ARA in plaques did not correlate. The contents of EPA in adipose tissue, plasma phospholipids, and plaques were all positively correlated with statistically significant coefficients of correlation between all compartments.

## Discussion

In this study, we analyzed the content of EPA and ARA in adipose tissue biopsies from subjects with peripheral artery disease and evaluated whether these fatty acids were associated with the expression of 5-lipoxygenase as well as the formation of LTB_4_ and cysteinyl leukotrienes in femoral plaques. Furthermore, we measured the content of ARA and EPA in both plasma and plaques in order to establish a possible biological pathway in which the content of ARA and EPA in adipose tissue could affect the 5-lipoxygenase pathway in the arterial wall.

Our major finding, was a statistically significant association between the content of ARA in adipose tissue and the expression of 5-lipoxygenase in atherosclerotic plaques, which persisted after adjustment for age, sex, and BMI. We also observed a weak positive correlation between adipose tissue ARA contents and LTB_4_ formation in plaques which, however, did not reach statistical significance (p = 0.14). Other investigators have previously assessed the expression of 5-lipoxygenase in atherosclerotic plaques [12-16] although none of them assessed femoral artery plaques. Two recent studies [13,15] have linked both 5-lipoxygenase expression and LTB_4_ formation to carotid plaque instability by reporting higher levels in unstable compared to stable plaques, as assessed by the presence of recent neurological symptoms. Other results supporting these findings were reported by Zhou et al. [12] who found that 5-lipoxygenase expression and LTB_4_ formation in carotid plaques were higher in diabetic compared to non-diabetic patients and by Spanbroek et al. [14] who found that 5-lipoxygenase expression was higher in more advanced atherosclerotic lesions. Although associations between the content of ARA and EPA in adipose tissue and 5-lipoxygenase pathway constituents in plaques have not been assessed previously, some indirect evidence for such an association has been derived from genetic studies. Dwyer et al. [17] thus reported that homozygous carriers of a short tandem repeat polymorphism in the promoter region of the gene encoding 5-lipoxygenase were significantly associated with a higher carotid intima-media thickness. This association was stronger for individuals with high dietary ARA intakes and attenuated for individuals with high dietary intake of marine omega-3 polyunsaturated fatty acids. In another genetic association study, Allayee et al. [18] was, however, unable to demonstrate an association between the same promoter polymorphism and the risk of myocardial infarction. However, subjects with high intakes of ARA had a significantly higher risk (OR = 1.32) of myocardial infarction compared to subjects with low ARA intakes (OR = 0.80). No gene-diet interaction was found for omega-3 fatty acids. In the present study, the finding of a positive association between the content of ARA in adipose tissue and the expression of 5-lipoxygenase in plaques is in line with the results of these nutrigenomic studies and thus provides further evidence for the importance of this fatty acid on the 5-lipoxygenase pathway. All together, these findings strengthen the hypothesis that the positive association between adipose tissue ARA and the risk of myocardial infarction [2-4] reported in epidemiological studies could be causal, at least partly, through higher expression of 5-lipoxygenase in the arterial wall.

Adipose tissue ARA was not correlated with either cysteinyl leukotriene formation in plaque or total body cysteinyl leukotriene formation, of which the latter can be estimated from urinary LTE_4_ levels [19]. While evidence for a link between LTB_4_ and plaque instability is supported by quite concordant results from several studies, the role of cysteinyl leukotrienes in plaque biology is more ambiguous. Qiu et al. [15] found a positive correlation between plaque instability and mRNA levels of the enzymes used for LTB_4_ synthesis (5-lipoxygenase, 5-lipoxygenase activating protein, LTA_4_ hydrolase) but not for cysteinyl leukotriene synthesis (LTC_4_ synthase). On the other hand, urinary LTE_4_ levels have been reported to be significantly higher in patients suffering from acute coronary syndrome compared to controls [20], and cysteinyl leukotrienes have been shown to be capable of inducing coronary spasm in ex vivo atherosclerotic coronary arteries but not in normal arteries [16]. Based on the results from the present study one could speculate that advanced femoral atherosclerotic plaques have a low expression of LTC_4_ synthase since 5-lipoxygenase expression was not significantly correlated to cysteinyl leukotriene levels in stimulated plaques.

We expected the content of EPA in adipose tissue to be associated with lower levels of leukotriene formation and 5-lipoxygenase expression in plaques, since dietary supplementation with fish oil (EPA + DHA) has been associated with increased stability of carotid plaques [21]. Furthermore, the content of EPA in plaques was associated with more stable and less inflammatory plaques [22]. Despite high correlations between EPA levels in adipose tissue, plasma phospholipids, and atherosclerotic plaques, suggesting a high degree of incorporation of dietary EPA into all these compartments, we actually found positive correlations between the content of EPA in adipose tissue and 5-lipoxygenase expression as well as LTB_4_ and cysteinyl leukotriene formation in plaques. These correlations were, however, non-significant, and hence no “harmful” association was detected per se, but surely, this study does not suggest any clinically beneficial association between EPA and the formation of 5-lipoxygenase products in femoral plaques. In a supplementary exploratory analysis, we determined correlation between the content of linoleic acid and docosahexaenoic acid in adipose tissue and 5-lipoxygenase expression and leukotriene formation in plaques, since these PUFA could potentially affect leukotriene formation in plaques. However, no significant correlations were observed.

**Figure 3:**
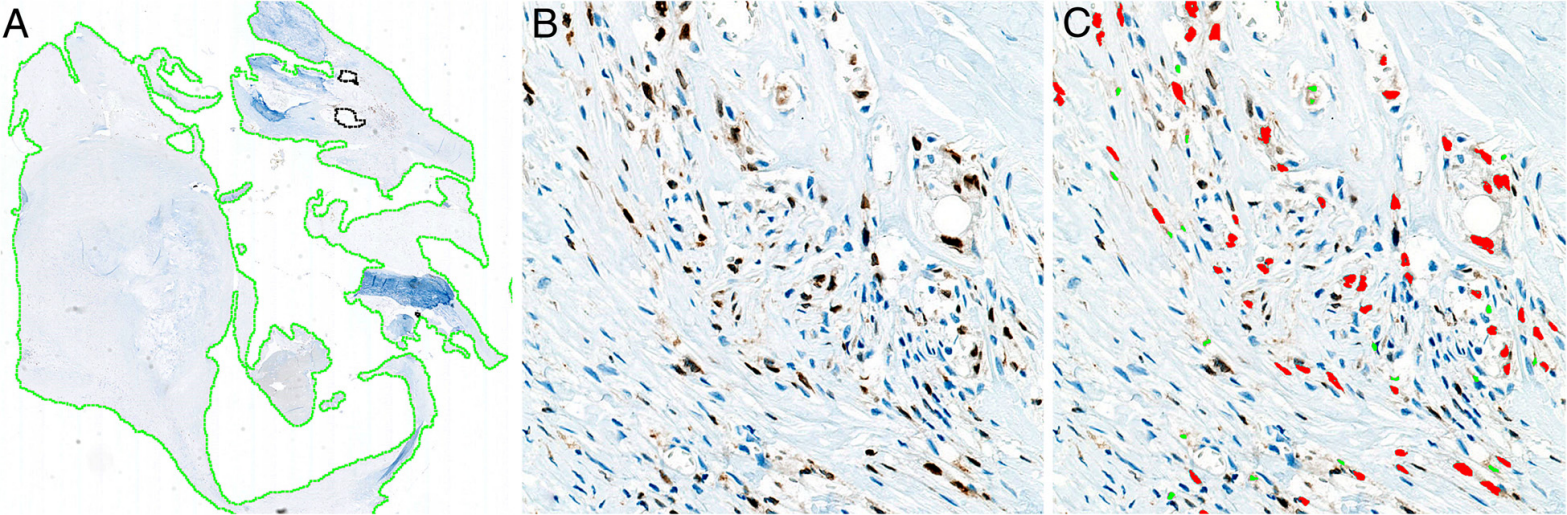
**Quantification of 5-lipoxygenase expression in plaques. A:** The section of the plaque is automatically outlined as the region of interest. **B:** Immunostaining of the plaque with monoclonal rabbit 5-lipoxygenase antibodies (zoom x 200). **C:** Automated detection of the stained areas using image analysis application software developed for 5-lipoxygenase staining.

In order to causally affect the 5-lipoxygenase pathway in the arterial wall, adipose tissue should be able to deliver increased amounts of ARA into the circulation and probably also onward into the arterial wall. This mechanism seems plausible, since ARA (and EPA) is far more likely to be liberated from adipose tissue compared to other fatty acids [23] indicating that even small differences in the content of these PUFA in adipose tissue could be clinically relevant. In the present study we found evidence that adipose tissue ARA is highly correlated with circulating ARA in plasma phospholipids (Figure 3). It should be acknowledged that fatty acids is released from adipose tissue as free fatty acids and not as phospholipids, however a previous study suggested that free ARA (non-esterified) in plasma is highly correlated with plasma ARA contents in phospholipids [24]. The content of ARA in plasma phospholipid was also correlated with the content of ARA in plaque, which makes it plausible that ARA originating from the adipose tissue could be incorporated into the arterial wall. However, no direct correlation was seen between the content of ARA in adipose tissue and the content of ARA in plaques in this study. This lack of correlation could be due to somewhat imprecise measurements of ARA in plaques (Coefficient of variation (CV) = 26%).

Several weaknesses of this study should be acknowledged. The selection of study tissue is open for discussion. We looked at femoral artery plaques which were in a chronic stage of fibrosis, calcification, and most often without any lipid core which is in contrast to carotid plaques [25]. The grade of inflammation in femoral plaques was lower than expected, making it harder to detect correlations between adipose tissue fatty acids and leukotriene formation/5-lipoxygenase expression in plaques. Furthermore, the extensive calcification in these plaques sometimes made them fall apart when they were dissected prior to stimulation. Only, coherent plaque tissue was weighed and stimulated (since calcium crystals would be inactive tissue), which could have introduced imprecision in the assessment of leukotriene formation from stimulated plaques, since they were expressed as ng leukotriene/g plaque. Furthermore, the formation of leukotrienes in plaques was measured after in vitro stimulation of plaques, which makes clinical interpretation of results more complex. Residual confounding cannot be excluded, since the size of the study only allowed us to include three potential confounders (age, sex, and BMI) in the multiple linear regression analyses. The main strength of the study is the accurate and reproducible assessment of the exposures of interest (adipose tissue ARA and EPA) and of 5-lipoxygenase expression in plaques.

## Conclusions

We found a positive association between the content of ARA in adipose tissue and the expression of 5-lipoxygenase in femoral atherosclerotic plaques, but no significant correlation with leukotriene formation was seen. The content of EPA in adipose was not significantly correlated with any of the 5-lipoxygenase pathway constituents. Both the ARA and the EPA content in adipose tissue was significantly positively correlated with their respective PUFA in plasma phospholipids, which again was correlated with the respective PUFA in plaques. This study provides evidence for a possible biological link between the content of ARA in adipose tissue and coronary heart disease. However, confirmatory studies assessing the association between the content of ARA in adipose tissue and the expression of 5-lipoxygenase pathway constituents in more inflammatory plaques (i.e. carotid artery) should be undertaken in order to explore this hypothesis further.

## Methods

### Subjects and design

In a cross sectional design, we included fifty consecutive subjects scheduled for femoral artery thrombendarterectomy due to symptomatic peripheral artery disease. The inclusion criteria were: age > 18 years and a diagnosis of peripheral artery disease with planned femoral thrombendarterectomy. Exclusion criteria were any one of the following: allergic asthma or rhinitis, any chronic inflammatory disease, systemic steroid treatment, cancer, acute coronary syndrome within the last 30 days, and inability to give informed consent. Patient history, medication, vital parameters, and food frequency questionnaires were recorded at admission, while blood and urine samples were obtained in the fasting state on the day of surgery. Atherosclerotic femoral artery plaques as well as adipose tissue biopsies from the subcutis of the thigh were collected during surgery. Approval of the study was obtained from the regional research ethics committee (N-20100047), and all patients gave informed consent.

### Analysis of atherosclerotic plaques

During femoral artery thrombendarterectomy, plaques were removed and placed in isotonic saline and immediately brought to the research laboratory together with the adipose tissue biopsy. Two small pieces of the plaque were cut off. The first piece was used for the analysis of leukotriene formation, while the other piece was used for analysing the content of ARA and EPA. The remaining part of the plaque was fixed in formalin followed by decalcification in formic acid.

Cross-sectioning of the plaque was performed at 3 mm intervals, and the resulting blocks were embedded in paraffin. From each block one 3 μm section was cut and stained with hematoxylin and eosin. An experienced consultant pathologist (MV) identified the histological section with the largest plaque area (culprit lesion) and did a descriptive histological evaluation of the atherosclerotic lesion in this section. Then, new 3 μm sections from this block and the two neighboring blocks were cut. We chose this area of the plaque since this is anticipated to contain the most inflammation [26,27]. All three sections were immunostained for 5-lipoxygenase, using anti-5-lipoxygenase monoclonal rabbit antibody clone C49G1 (Cell Signaling Technology, Inc. USA). An irrelevant monoclonal rabbit antibody in the same concentration as the 5-lipoxygenase antibody was applied as negative control on representative slides and showed no staining reaction. For image acquisition (Figure 3), the immunostained slides were scanned on a nanozoomer HT 1.0 (Hamamatsu, Japan) with the 40× source lens setting and then loaded into image analysis application software developed for 5-lipoxygenase staining (Visiopharm, Denmark). The percentage of the plaque area occupied by 5-lipoxygenase for each section was then calculated automatically by the computer software and the average staining fraction from the three sections was used as the primary outcome. The intra-observer CV was 0% based on a re-evaluation of ten sections after 3 months.

The piece of plaque used for leukotriene analysis was cut into smaller pieces and put into stimulation vials. After stimulation with calcium ionophore A23187 the levels of LTB_4_ (R&D Systems, Minneapolis, USA) and cysteinyl leukotrienes (Enzo Life Sciences Inc, New York, USA) were quantified using solid phase immunoassay ELISA kits with a competitive technique. To adjust for inter-individual differences in the amount of plaque tissue, the levels of leukotrienes were expressed as ng/g plaque tissue. The intraserial CV was 5% for LTB_4_ and 4% for cysteinyl leukotriene measurements.

Lipids of the plaque were extracted according to Folch et al. [28] and the fatty acid composition was analyzed by gas chromatography as earlier described [11]. The content of ARA and EPA was expressed as percent of total fatty acids and the intraserial CV was 26% for both fatty acids.

### Analysis of the content of PUFA in adipose tissue

Adipose tissue biopsies from the subcutis of the thigh were obtained during surgery and placed in dry vials. Approximately 2–4 mg was analyzed for the content of individual PUFA using gas chromatography and the intraserial CV was 4% for ARA and 8% for EPA.

### Analysis of the content of PUFA in plasma phospholipids

Fasting blood samples were collected from all patients on the day of surgery. In the assessment of the individual fatty acid contents in plasma phospholipids, total lipids were extracted from plasma according to Folch et al. [28]. Separation of fatty acids from plasma phospholipids was done according to Burdge et al. [29]. The fatty acid composition was analysed by gas chromatography and the intraserial CV was 1% for ARA and 2% for EPA.

### Leukotriene E_4_ in urine

Quantification of urine LTE_4_ levels was done using a solid phase immunoassay ELISA kit (USCN life science Inc, Wuhan, China) and the intraserial CV was 17%.

### Statistics

We used Pearson’s product–moment coefficient of correlation model to explore associations between our exposures (adipose tissue EPA and ARA) and the primary outcomes (5-lipoxygenase, LTB_4_, cysteinyl leukotrienes in plaques) and secondary outcomes (urinary LTE_4_ levels, EPA and ARA in plasma phospholipids, EPA and ARA in plaques). We further explored associations with our primary outcomes using multivariable linear regression models, in which we included the following a priori selected potential confounders: Age (continuous), sex (dichotomous), and BMI (continuous). In exploratory analyses, we also evaluated adipose tissue linoleic acid and docosahexaenoic acid as exposure variables. The distributions of most of the variables were positively skewed, and logarithmic transformation was therefore applied when appropriate. Assumptions of normality, linear relationship, and equal variances were found to be satisfactory when assessed by relevant graphical plots. Statistical tests were two-tailed, and P < 0.05 was considered significant. The STATA 11 software package (Stata Corp., College Station, TX, US) was used for all analyses.

## Abbreviations

ARA: Arachidonic acid; CV: Coefficient of variation; EPA: Eicosapentaenoic acid; LTB_4_: Leukotriene B_4_; PUFA: Polyunsaturated fatty acid.

## Competing interests

The authors declare that they have no competing interests.

## Authors’ contributions

The authors’ responsibilities were as follows: MN, MLMG, MV, KO and EBS: Developed the study design and supervised the study; MN and EBS: Obtained funding; MN, MLMG, MV and AA: Collected materials and data; MV: Analyzed all immunostained sections of the plaque; MN and KMD: Conducted the statistical analysis; MN, KO, KMD and EBS: Analyzed and interpreted data; MN: Drafted the manuscript, including figures and tables; MLMG, MV, AA, KO, KMD and EBS: Contributed to the critical revision of the manuscript. All authors read and approved the final manuscript.
